# Upgraded front ends for SLS 2.0 with next-generation high-power diaphragms and slits

**DOI:** 10.1107/S160057752400907X

**Published:** 2024-10-22

**Authors:** David Marco Just, Marcel Brüstle, Simon Guntli, Johannes Mück, Claude Pradervand

**Affiliations:** a Paul Scherrer Institute PSI Forschungsstrasse 111 5232Villigen PSI Switzerland; Advanced Photon Source, USA

**Keywords:** front end, photon beamlines, SLS 2.0, synchrotron upgrade, beam mask, diaphragm, high-power slits, photon shutter, vacuum window, X-ray beam position monitors, compact footprint, high-power load

## Abstract

The upgrade of the Swiss Light Source, called SLS 2.0, necessitates comprehensive updates to all 18 user front ends. This upgrade is driven by the increased power of the synchrotron beam, reduced floor space, changing source points, new safety regulations and enhanced beam properties, including a brightness increase by up to a factor of 40. While some existing front-end components are being thoroughly refurbished and upgraded for safety reasons, other components, especially those designed to tailor the new synchrotron beam, are being completely rebuilt. These new designs feature innovative and enhanced cooling systems to manage the high-power load and meet new requirements such as mechanical stability and compact footprints.

## Abstract 2

This paper describes the front ends and their components for the upgrade of the Swiss Light Source. Parts of the existing front ends are being refurbished while other components are completely new with enhanced cooling capacity and mechanical stability.

## 1. Introduction

SLS 2.0 is the upgrade program of the Swiss Light Source (SLS) synchrotron at the Paul Scherrer Institut (PSI) in Villigen near Zürich in Switzerland. The goal of this upgrade is to increase the brightness of the X-ray beams by up to a factor of 40 (Braun *et al.*, 2021 ▸) by transforming the existing SLS (in operation from 2001 to 2023) from a third-generation facility to a diffraction-limited storage ring (DLSR) fourth-generation synchrotron, with a new magnet lattice. To achieve this, the old storage ring is being replaced within the existing building from October 2023 to December 2024. The electron-beam energy will be increased from 2.4 to 2.7 GeV; the electron-beam current remains at 400 mA. The new storage ring features a multi-bend achromat design, including antibends, and has a threefold symmetry.

The front end transports the synchrotron beam, generated by insertion devices, bending magnets and superbends in the storage ring, through the concrete shielding tunnel to the 18 user beamlines of SLS 2.0. The primary stopper, which is part of the personnel safety system, allows for safe blocking of radiation to enable work in downstream areas of the beamline. Fixed-beam apertures (so-called diaphragms), slits, vacuum windows and filters define and condition the synchrotron beam, while X-ray beam position monitors (XBPMs) provide feedback to both the machine and the beamline users. Along with the vacuum windows, fast and gate valves protect the storage-ring vacuum from air inrushes and poor vacuum conditions upstream of the beamline.

### 1.1. Upgrade strategy

At SLS 2.0 we will install a total of 18 front ends for user beamlines. For various logistical reasons, the installation of these front ends is divided into two phases. The 12 front ends in Phase 1 will be installed in Q4 2024 and commissioning is planned to start in Q2 2025. The vacuum port of the remaining six front ends will be closed with a fixed absorber until the front ends are installed in Phase 2 in Q1 2026 and go into user operation in Q3 2026. A complete list of all user front ends, along with their respective installation phases, can be found in Table 1 ▸. A graphical overview of the energy range and the scientific focus for each user beamline can be found in Appendix *A*[App appa].

In addition to Phase 1 and Phase 2, we installed two bending-magnet front ends (X01DA and X06DA) in summer 2023, in a phase referred to as Phase 0. This early installation allowed us to test new concepts for the SLS 2.0 front ends before the major upgrade in Phase 1. Although these new front ends needed to be removed from the tunnel and modified during the dark time to fit the new storage ring, the feedback from these tests was of great value. Even more crucial was the experience we gained in collaboration and logistics during the installation and commissioning phases with various teams, such as the electricians, the controls, water, alignment and vacuum groups. This experience helped us identify areas where more time was needed and where requirements and coordination could be improved, which was, along with the on-the-job training of the various technicians, very valuable for the ongoing upgrade of the Phase 1 front ends (Just *et al.*, 2024 ▸). A comparison of the layout of the PX-III (X06DA) front end in Phase 0 and Phase 1 as well as in its original SLS state can be found in Appendix *B*[App appb].

Despite the increased complexity of ordering, refurbishment and installation, we decided to upgrade the existing SLS front ends for SLS 2.0 rather than ordering all components entirely new. This decision was driven not primarily by cost and effort reduction but by the desire to maintain a proven, reliable concept wherever external factors such as power load, floor space and mechanical stability requirements allowed it. This concept also helped to reduce commissioning efforts and significantly enhanced overall sustainability. We are reusing all vacuum components (ion getter pumps, vacuum gauges and vacuum valves) after successful functionality tests without modifications. However, all other components were either designed completely new or modified to meet new requirements. The primary stoppers were upgraded to comply with new safety regulations, cycled motion bellows were replaced with new ones with a lifespan well above that expected for SLS 2.0, and new diaphragms and slits were designed to match the new beam sizes due to the smaller emittance of the storage ring and changes in the X-ray sources. Additionally, we developed novel cooling strategies for these devices to handle the increased thermal load and mechanical stability, replaced all relative with absolute encoders, upgraded the readout and control electronics, modified the geometry of our XBPMs and designed new elements with a focus on compactness.

#### 1.1.1. Compact footprints

The new lattice of the storage ring reduces the available floor space for the front ends and changes all source points of the beamlines. Therefore, the updated front ends need to be more compact than those from the SLS era. One way to address this was by combining functions. For example, in bending-magnet and superbend front ends we eliminated the use of a fixed mask or diaphragm and instead use slits to define the maximum beam size and power on downstream elements. We also designed compact supports and generally smaller devices.

**Figure 1:**
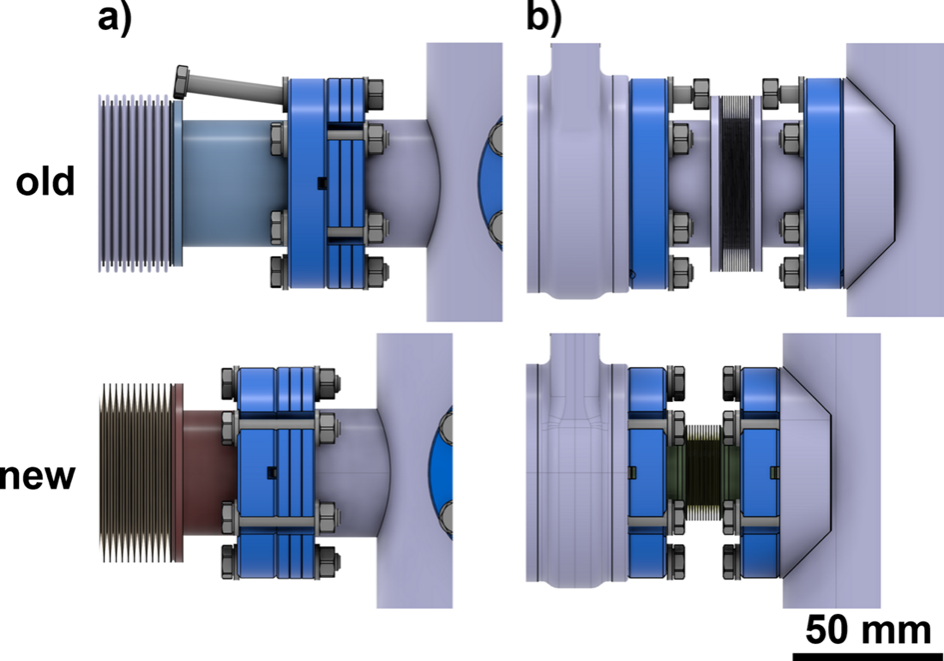
Floor-space reduction at component interconnections. Top: conventional interconnections. Bottom: new, compact interconnections. (*a*) The use of slotted flanges reduces the distance between flanges and adjacent elements by allowing screws to be inserted through the slots. (*b*) Bellows with a smaller diameter, combined with slotted flanges, minimize the distance between two elements, achieving a separation of only 50 mm for two DN40CF elements.

Additionally, special attention was given to the flanges and bellows used. Since most flanges must be equipped with screws and nuts, the screws at the original SLS needed to be inserted from one side of the flange. Consequently, the space between the flange and any element needed to be slightly larger than the screw. For the SLS 2.0 front ends, we opted for slotted flanges instead. This design minimizes the distance between a flange and an element since the screw can be inserted through the slot [see Fig. 1 ▸(*a*)]. In other cases, we reduced the bellows size for a given flange. For instance, a bellows that is normally used with DN16CF flanges was paired together with DN40CF flanges, which allows for shorter interconnections between elements [see Fig. 1 ▸(*b*)].

#### 1.1.2. Thermal management

With the increase in storage-ring energy and the introduction of new sources, both the power and power density that the front ends must handle have increased by up to a factor of three (Just & Pradervand, 2021 ▸). To manage this increase, we analysed each component exposed to the synchrotron beam using computational fluid dynamics and finite element analysis with *ANSYS CFX* (*Fluid Flow*) and *ANSYS Mechanical*.

All components directly exposed to the synchrotron beam are made from oxygen-free copper (Cu-OFE, CW009A). The criteria determining whether a component can withstand the thermal load or not are based on the APS-U design criteria (Grudzinski *et al.*, 2020 ▸) and are summarized as follows:

(i) The average velocity inside a water-cooled cooling channel must be below 2 m s^−1^.

(ii) The wall temperature of a cooling channel must be below the boiling temperature of water at the pressure of the return line. In a conservative approach we used 100°C as the limit.

(iii) The surface temperature of all copper devices exposed to thermal load must be below 200°C.

We established that the existing photon absorbers of the primary stopper can withstand all load cases. This allowed us to reuse these devices for SLS 2.0, resulting in a substantial reduction of costs and engineering effort. For new designs, we established a set of design rules to minimize thermal expansion and enhance the cooling capacity based on the formula for thermal conduction of a plate,
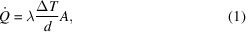
where 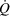 is the thermal conductance, *i.e.* the amount of heat transported per time unit, λ is the specific thermal conductivity, Δ*T* is the temperature difference between the warm and the cool side, *d* is the distance between the warm and the cool side, and *A* is the active surface area of the heat conductance. Consequently:

(i) The cooling channels should be as close as possible to the surface exposed to the heat load since the heat conduction is inversely proportional to this distance *d*.

(ii) The distance *d* of the cooling channel from the exposed surface should not be less than 3 mm. This ensures mechanical stability and prevents leakage due to cavitation or chemical corrosion.

(iii) To maximize the active cooling surface *A*, there should be as many cooling channels as possible, or the thermally exposed surface should be completely surrounded by water.

(iv) The cooling water inlet should be as close as possible to the warmest part of the thermally exposed device to maximize Δ*T*.

## 2. Front-end layout

At SLS and SLS 2.0, each front end is unique for several reasons. The existing front ends were produced and installed over approximately eight years (1999–2007), during which the designs of the individual components and the front-end layouts evolved. Additionally, the ‘dog legs’, the straight, tangential parts of the ring tunnel where the front ends are located, vary in length. Each beamline has specific design requirements for its front end, in particular the selection of a specific beam acceptance (see Table 1 ▸), tailored to match the source and the scientific purpose of the beamline. Some beamlines also request specific components, such as filters.

For SLS 2.0, the front ends can be divided into two families, the bending-magnet front ends (including superbends) and the insertion-device front ends. These families differ mainly in their ability to handle and absorb high heat loads. Each family is further divided into two subgroups: hard X-ray (HXR) and soft X-ray (SXR) front ends. While HXR beamlines typically do not require measurements of photon energies below ∼2 keV, soft X-ray beamlines focus primarily on lower energy ranges between approximately 15 eV and 2 keV. Since photons at these energies even interact with residual gases, SXR front ends are designed without vacuum windows and all components must be baked-out. In contrast, HXR front ends are equipped with a vacuum window and bake-out is only necessary up to this window.

### 2.1. Insertion-device front ends (IDFEs)

**Figure 2:**
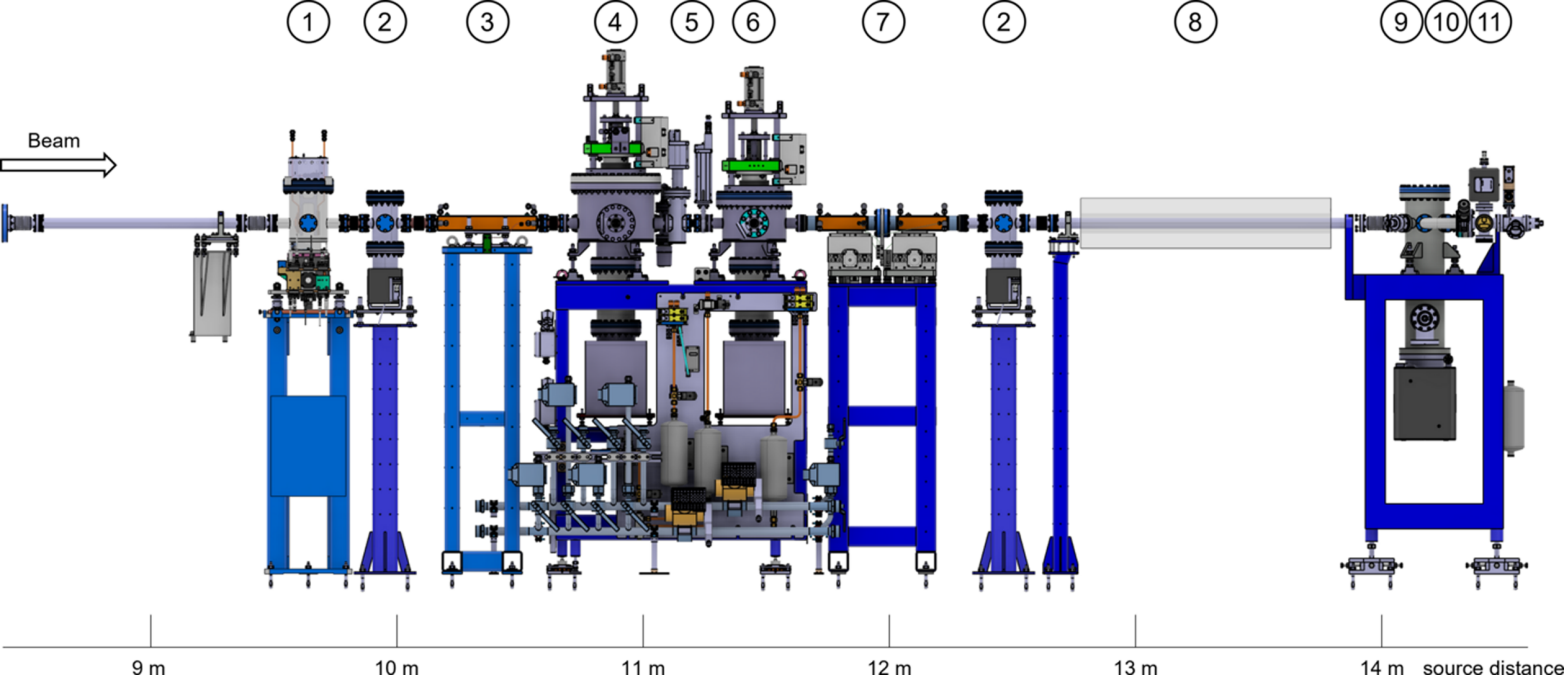
A typical hard X-ray insertion-device front end (IDFE), here X10SA (PX-II). (1) XBPM, (2) SLS 2.0 standard pump stand, (3) high-power diaphragm, (4) photon shutter, (5) gate and fast valve, (6) beam stopper, (7) high-power slits, (8) tunnel wall, (9) existing (SLS) after wall pump stand, (10) vacuum window with bypass, (11) front-end terminating gate valve. The photon shutter (4) and the beam stopper (6) function together as the primary stopper.

A typical hard X-ray IDFE is shown in Fig. 2 ▸. A typical soft X-ray IDFE would be comprised of the same components except for the vacuum window (position 10 in Fig. 2 ▸).

#### 2.1.1. X-ray beam position monitors (XBPMs)

The XBPMs provide feedback regarding the X-ray beam position to both the machine and the beamline users. We plan to integrate their signal into the slow orbit feedback system of the storage ring. In a later phase, we also aim to use the XBPMs for fast orbit feedback.

The previous IDFEs at SLS were equipped with two tungsten blade XBPMs. The first was positioned at the very beginning of the front end, similar to the planned positions for SLS 2.0; the second was located after the primary stopper.

For SLS 2.0, we decided to install only one XBPM per IDFE for several reasons. First, the layout in Fig. 2 ▸ shows that there is no available floor space between the primary stopper and the tunnel wall for an additional device, making the installation of a second XBPM challenging. Additionally, since the second XBPM would be located behind the primary stopper, which is operated solely by the beamline, its signal availability to the storage-ring feedback loops is limited, and it cannot be used permanently to steer the electron beam. Cost considerations also played a role in this decision.

**Figure 3:**
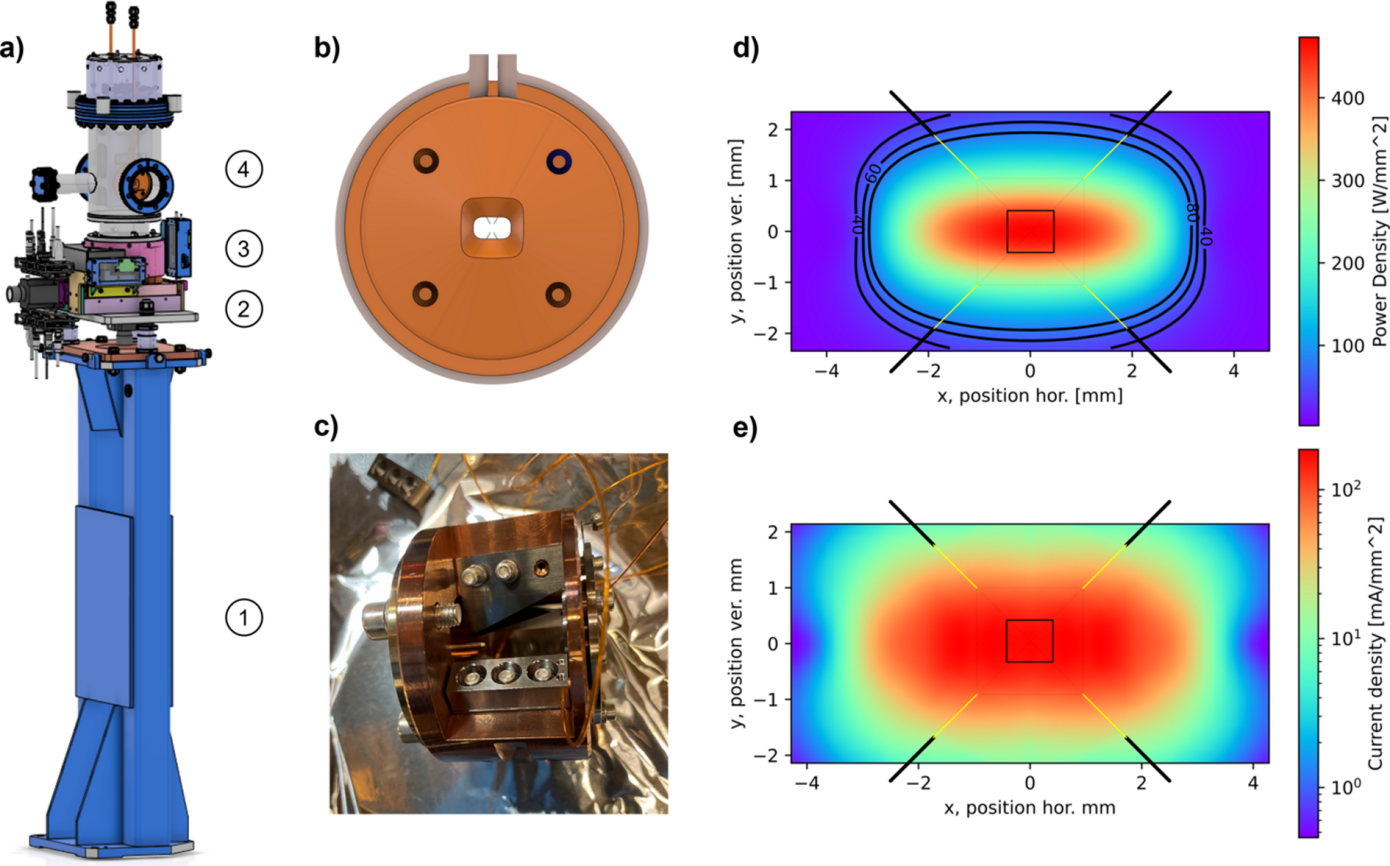
X-ray beam position monitor (XBPM). (*a*) 3D model of an XBPM at SLS 2.0. IDFEs featuring: (1) support, (2) motion stage, (3) encoders and (4) vacuum chamber. (*b*) Detail showing the 0.2 mm-thick tungsten blades behind the water-cooled copper shielding. (*c*) Photograph of a partially disassembled blade holder. (*d*) Power-density plot and (*e*) current-density plot, both indicating the new blade position with solid black lines. The solid yellow lines indicate the blade position without changes (*i.e.* SLS configuration). The field of view in both plots represents the synchrotron beam at the position of the XBPM, while the small rectangle in the centre corresponds to the beam used by the beamline (*i.e.* after the diaphragm).

The XBPMs for SLS were manufactured by FMB Berlin [see Fig. 3 ▸(*a*)]. For SLS 2.0, we decided to reuse almost all components. We dismantled the motion stage, cleaned and lubricated all the spindles and guides, replaced the existing incremental encoders with absolute encoders and replaced all motion connectors to fit the new EtherCAT-based motion-control system.

Using a reverse engineering approach, we calculated the power distribution for the existing SLS XBPMs, estimating that the maximum power density on the blades is approximately 70 W mm^−2^, with total power absorption of less than ∼50 W. This estimate was verified by Karsten Holldack from the Helmholtz-Zentrum Berlin, who calculated the blade position for the XBPMs of SLS.

Using *Spectra* (Tanaka, 2021 ▸) we calculated the power density and the current density for each undulator and XBPM position [see Figs. 3 ▸(*d*) and 3 ▸(*e*)]. Fig. 3 ▸(*d*) shows that maintaining the existing configuration (yellow lines) would exceed acceptable boundaries. Therefore, we needed to rearrange the blades to meet the power absorption requirements. For the new blade position, the nominal current on each blade will be in the range of 1–2 mA, which is suitable for our readout electronics. In most cases the new blade configuration could not be achieved with the existing blades. Consequently, we sent the complete blade holder [Fig. 3 ▸(*c*)] to FMB Berlin where the blades and the ceramic insulators were replaced with new components.

Mainly for economic reasons we decided to keep the existing LoCuM-4 current amplifiers from ENZ Berlin and connect them to our A/D convertor BECKHOFF EL3164, which is part of the new motion-control system. To start off with, this configuration is well suited for the slow feedback of the storage ring; for the use of the XBPMs with the fast orbit feedback, a change of the readout electronics may be required in the future.

#### 2.1.2. SLS 2.0 standard pump stand

**Figure 4:**
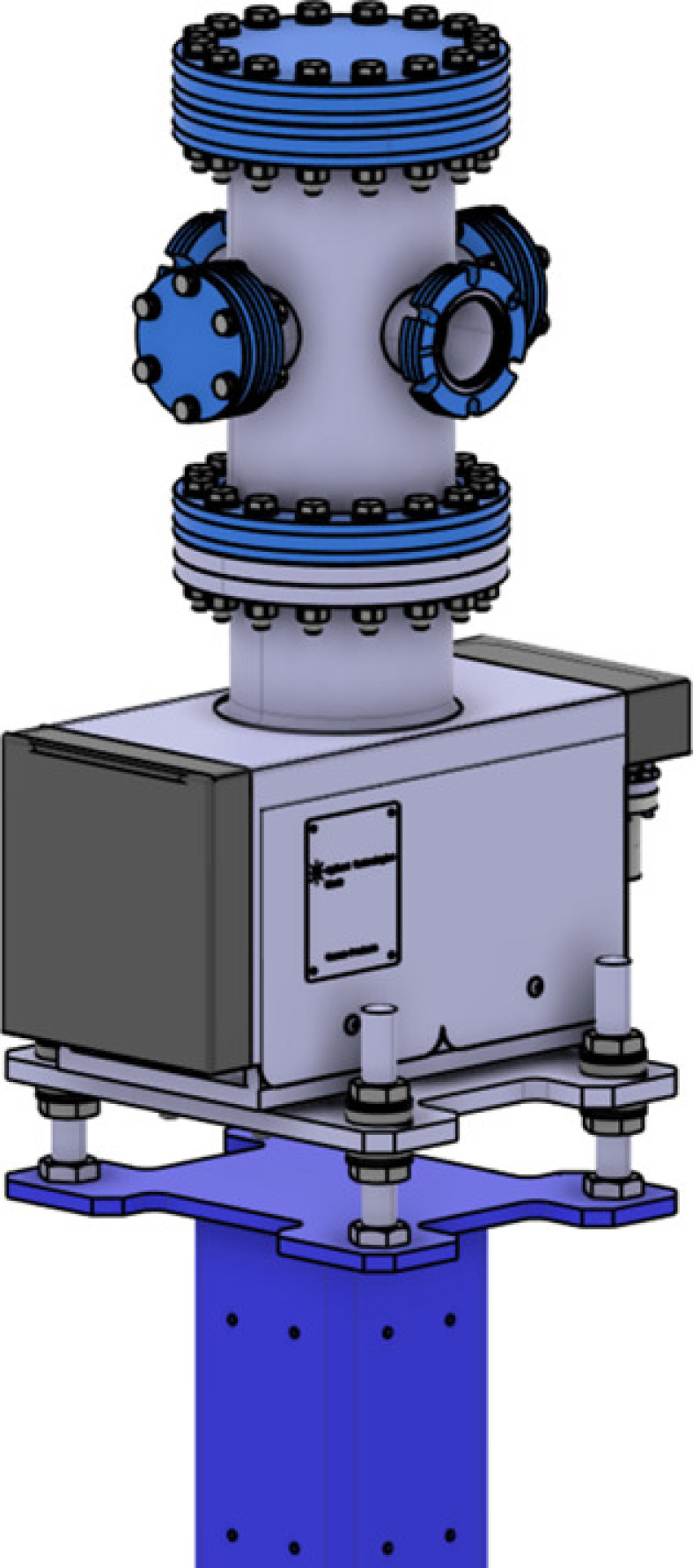
Standard pump stand for SLS 2.0 front ends with slotted flanges and ion getter pump directly mounted to the support.

Pump stands are used to maintain ultra-high-vacuum (UHV) conditions (typically better than 10^−9^ mbar) near components that are not equipped with vacuum pumps. Fig. 4 ▸ shows a standard pump stand for SLS 2.0 front ends. Unlike at SLS, where various pump stands with different ion getter pumps were used, we aimed to standardize all pump stands with a one-fits-all approach.

The design uses an off-the-shelf vacuum chamber from VAb Vakuum-Anlagenbau GmbH, made from 1.4429 stainless steel (316 LN). This chamber was modified to include two slotted flanges to save floor space and allow neighbouring components to be positioned as close as possible to the pump stand.

Although in most cases only two DN40CF flanges are required for the beam to pass through, all chambers are equipped with four DN40CF flanges and a free DN100CF flange. This design allows the chamber to be equipped with other components such as vacuum gauges or additional diagnostics and provides flexibility for future use, while maintaining the interchangeability and simplifying the ordering process.

We use existing 75 l s^−1^ Agilent Diode ion getter pumps, which were previously used in the storage ring. The pump is mounted on a simple kinematic mount, which is attached to a straightforward support. This support has a compact footprint and is equipped with numerous M6 threads for mounting cable trays, bakeout boxes, or other peripheral equipment.

#### 2.1.3. High-power (HP) diaphragm

The HP diaphragm, or fixed aperture, is used to define the beam size usable by the beamlines and to limit the power on downstream elements. For SLS 2.0, IDFEs needed new diaphragms to accommodate the increased power load and smaller beam sizes associated with the increased brightness of the storage ring, and the new and more powerful insertion devices.

**Figure 5:**
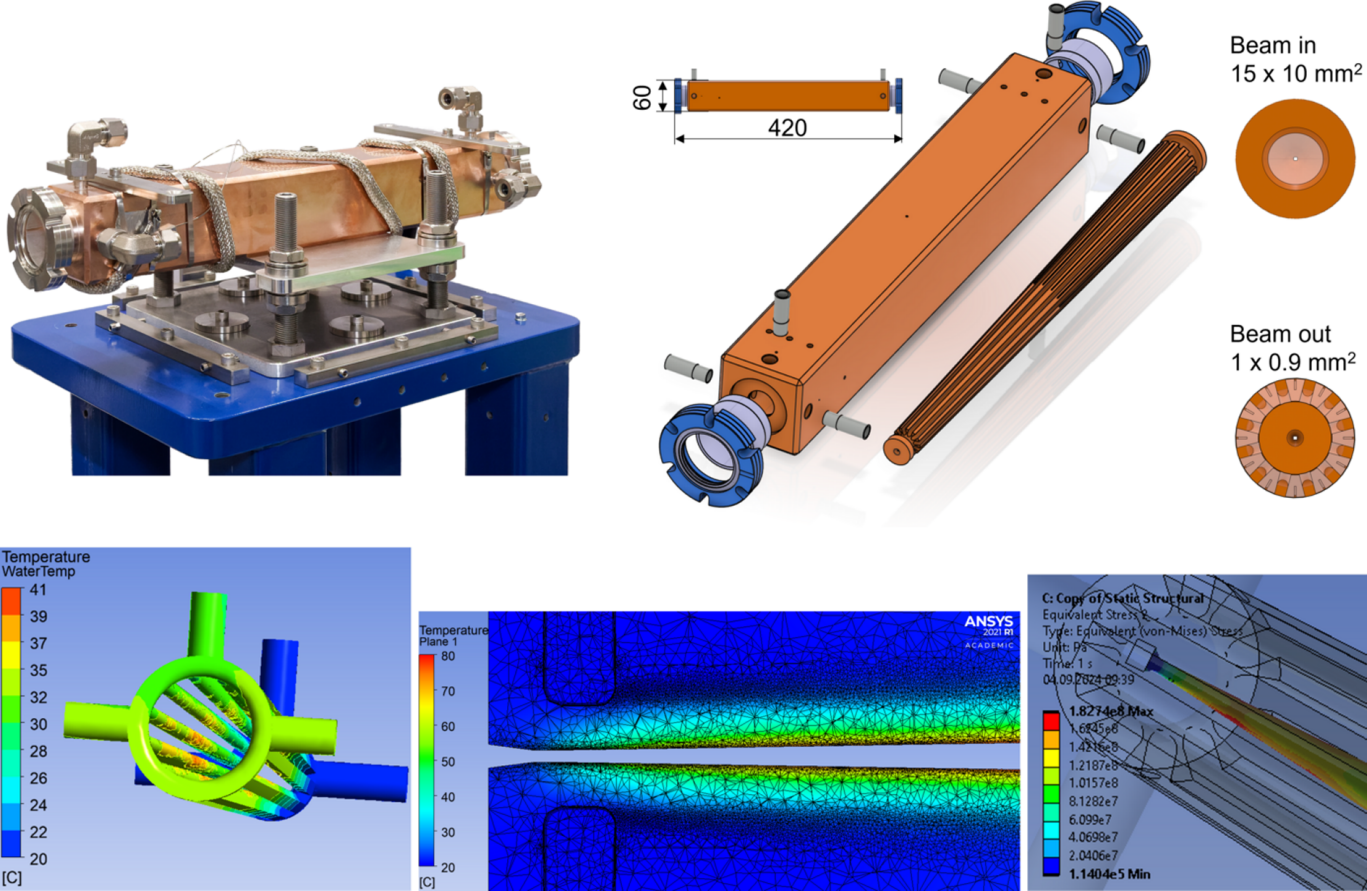
New high-power (HP) diaphragm. Top row. Left: photograph of an assembled HP diaphragm. Right: 3D model of the design and an illustration of the dimensions. The inlet is shaped in an elliptical form (15 mm × 10 mm) to avoid corners and edges which would cause thermal stresses. The exit window is a rectangular opening of only 1 mm × 0.9 mm (H × V), machined into the copper. Bottom row. Finite element analysis of the HP diaphragm at 15 kW power load and power density of 690 W mm^−2^ at 10 m source distance, showing excellent thermal behaviour: the maximum water temperature is lower than 45°C and solid temperatures are just below 80°C, while the maximum stress (Von Mises) in the beam channel is kept at around 180 MPa.

Fig. 5 ▸ shows the new design of the HP diaphragm. All IDFEs are equipped with a HP diaphragm of the same design, which are adapted to the individual beam size of each beamline (see Table 1 ▸). The design consists of an inner part and an outer sleeve, both made from oxygen-free high-conductivity copper (Cu-OFE) and joined with vacuum brazing. Together, they form 12 cooling channels which closely follow the tapering of the beam channel. To enhance cooling efficiency, each web between the cooling channels on the inner part is machined with a groove which is filled with Palcusil 10 before brazing, thus maximizing the thermal connection between the inner part and the outer sleeve. These 12 cooling channels, along with the three inlets and outlets, allow the HP diaphragm to be homogeneously cooled with 18 l min^−1^, while keeping the average velocity of the cooling water below 2 m s^−1^.

Before the inner and outer part were brazed together, a 0.7 mm-diameter hole was drilled through the entire 392 mm length of the inner part using sinker electrical discharge machining (EDM). After brazing, the beam channel was formed by wire erosion. The beam channel has an exit window as small as 1 mm × 0.9 mm and features an elliptical entrance window that transitions to a rectangular exit window, avoiding sharp edges and corners. This design choice is crucial for preventing local strain and stress forces due to thermal expansion. The beam channel distributes the thermal load along the entire length of the HP diaphragm, enabling it to handle the high power of the new sources. Fluid–solid heat transfer and mechanical stress/strain finite element analyses have demonstrated excellent performance for thermal loads of 15 kW and orthogonal power densities of 690 W mm^−2^ and beyond (Just *et al.*, 2022 ▸).

#### 2.1.4. Primary stopper

**Figure 6:**
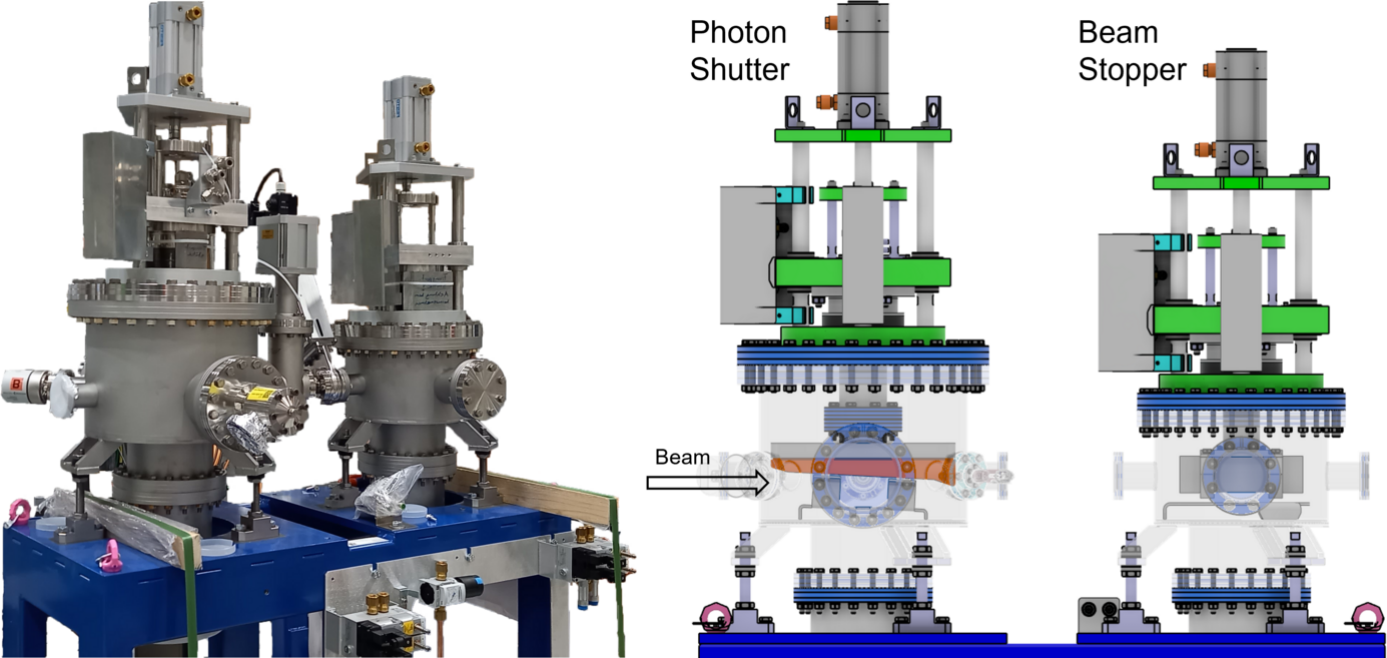
Primary stopper for IDFEs. Left: photograph of an assembled primary stopper. Right: 3D model of the design and differentiation of the photon shutter with the copper photon absorber (orange) used to absorb the high heat load synchrotron radiation and the beam stopper with the tungsten alloy absorber (grey cuboid) used to absorb high-energy *Bremsstrahlung*.

The primary stopper is the major safety element of the front end. When closed, it blocks all harmful radiation, allowing beamline personnel to work safely in downstream areas outside the ring wall. For insertion-device beamlines, the primary stopper consists of two elements, both mounted on the same support (see Fig. 6 ▸).

The first element is the photon shutter, which includes a water-cooled photon absorber made from copper (Cu-OFE). This shutter is pneumatically driven into the beam to absorb the thermal load generated by the synchrotron radiation. The second element is the beam stopper, a tungsten alloy cuboid (52 mm × 80 mm × 180 mm; H × W × L) made from Densimet G18 or equivalent. This stopper is also pneumatically driven into the beam to absorb the remaining high-energy radiation, such as *Bremsstrahlung*.

Both the photon shutter and the beam stopper are reused from the existing SLS front ends but have been substantially modified for SLS 2.0. We replaced all the motion bellows with ones designed to last for 1 million cycles. All pneumatic components have been upgraded to state-of-the-art versions to ensure long-term reliability, and new supports were built to reduce floor-space usage.

To improve safety and reliability, several enhancements were made. Existing micro-switches were replaced with certified safety end switches from two different manufacturers (diversity), mounted on separate supports (redundancy). The pneumatic control block was replaced with one certified for safety applications. Additionally, a fall-through protection mechanism, which holds the absorbers inside the beam in case of driving rod failure, was integrated into the design.

**Figure 7:**
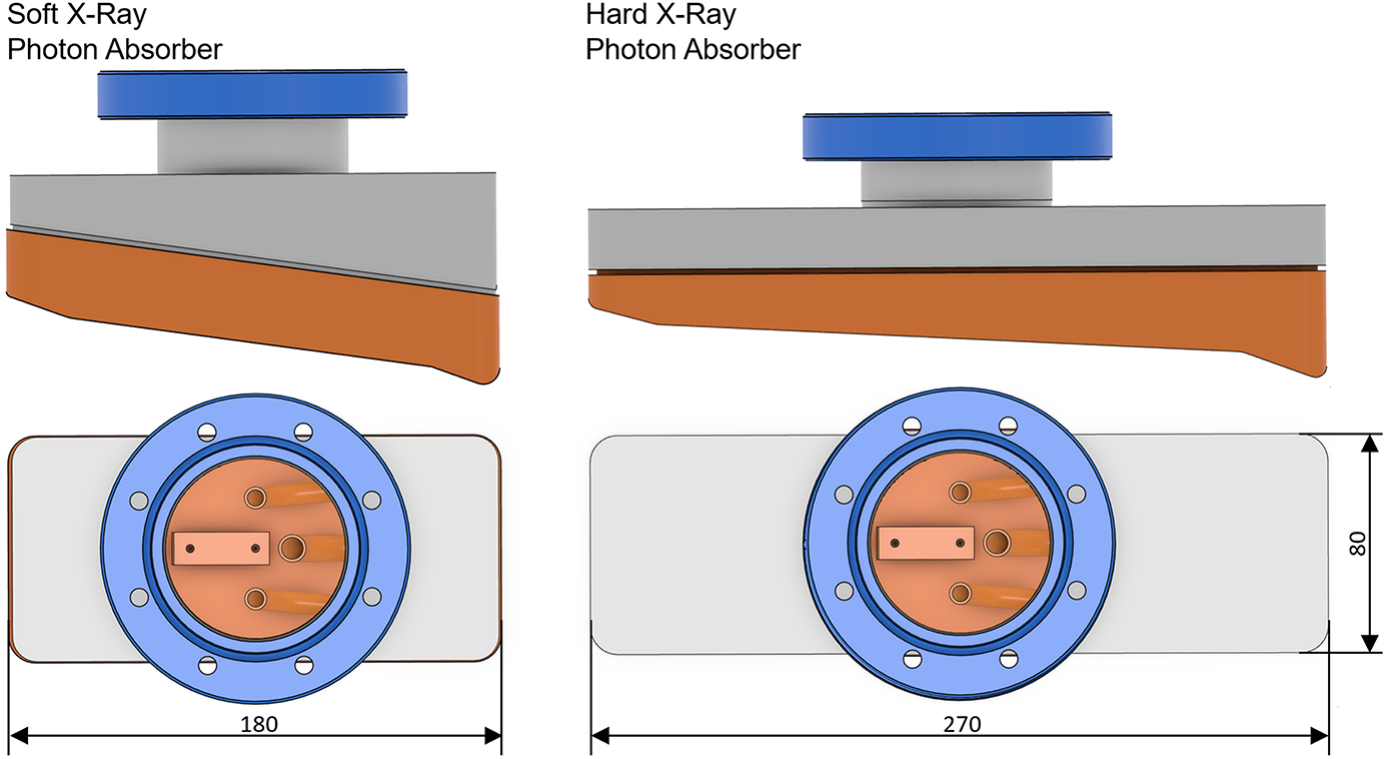
3D model of the two photon absorbers used in the photon shutters. Left: soft X-ray photon absorber. Right: hard X-ray photon absorber.

Depending on whether the photon shutter is used in a hard or soft X-ray front end, two different designs are employed (see Table 1 ▸). For soft X-ray front ends, the absorbers are shorter and steeper, optimized for larger beam sizes. In contrast, hard X-ray absorbers are longer and optimized for higher power densities, providing better load distribution due to the smaller incident angle (see Fig. 7 ▸).

#### 2.1.5. Gate and fast valve

Between the photon shutter and the beam stopper, a gate valve and a fast valve, both purchased from VAT, are installed. These valves are designed to separate and protect the ring vacuum in case of an air inrush. When a pressure rise is detected by the fast-valve sensor, located on the after-wall pump stand (see Fig. 2 ▸), the fast valve closes within 8 ms, roughly the time it takes for a pressure wave to travel the distance between the two elements at the speed of sound. Simultaneously, the photon shutter is triggered to close, as the fast valve alone cannot withstand the synchrotron beam. Finally, the gate valve, positioned upstream of the fast valve, also closes because the fast valve is not entirely UHV-tight. These valves, along with all other vacuum components, are reused from the existing SLS equipment.

#### 2.1.6. High-power (HP) slits

All IDFEs are equipped with new HP slits. The purpose of these slits is to reduce the beam size to fit the scientific case and to minimize thermal load on downstream elements, typically mirrors and monochromators. Additionally, they allow one to raster scan the beam using a small aperture and thus obtain the beam profile (pinhole scan).

**Figure 8:**
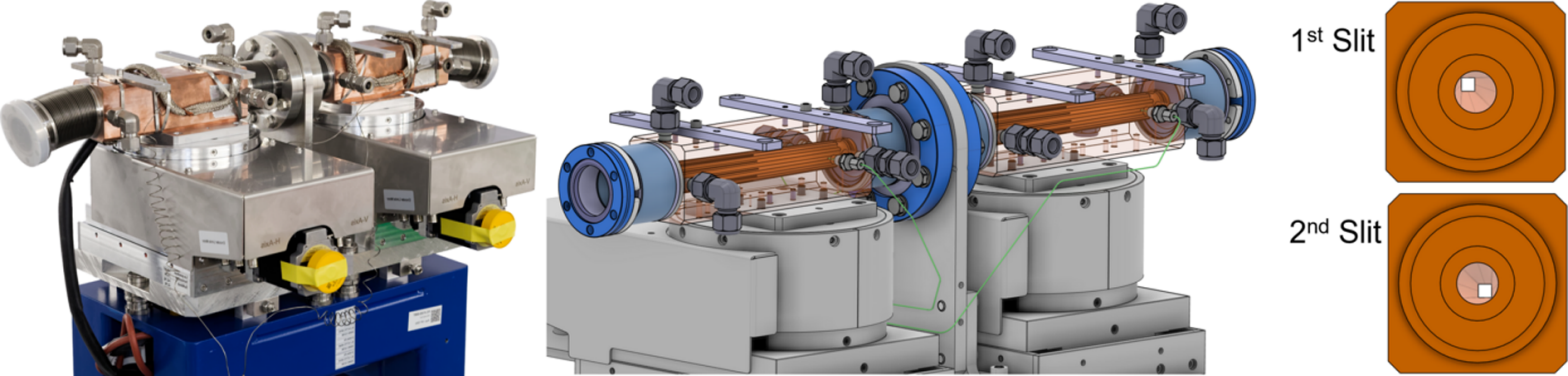
New SLS 2.0 HP slits. Left: photograph of the assembled HP slits ready for installation. Middle: 3D model of HP slits showing construction details. Right: front view of the two slits. The first slit trims the lower and right part of the beam, while the second slit trims the upper and left part.

The slits function together as what has also been referred to as ‘L-Slits’ (Chen *et al.*, 2008 ▸; Oura *et al.*, 1998 ▸; Shu *et al.*, 1995 ▸; Volpe *et al.*, 2017 ▸). The first slit trims the lower and right part of the synchrotron beam, while the second slit trims the upper and left part (see Fig. 8 ▸). By overlapping their projections, they allow for the adjustment of any desired aperture at any position, including fully closing the beam. In the fully opened position, the full, unclipped, beam can pass through.

One of the key advantages of this design is its compactness, as it eliminates the need for a separate vacuum chamber. Unlike the conventional design, which requires four individual blades to be positioned in the beam, this approach mounts the two slits directly onto the motion stage. This direct mounting significantly enhances mechanical stability, avoiding the issues seen in conventional designs where blades are extended through a chamber on long arms that amplify vibrations. Additionally, small grazing angles are used to increase the surface area exposed to the beam, effectively reducing the thermal load on each slit. This reduction in thermal load minimizes thermal expansion, further improving the overall mechanical stability of the slit system.

The design is derived from the HP diaphragm, featuring the same advanced cooling and stability properties. It includes an inner part and an outer sleeve forming 12 cooling channels, which closely follow the tapering of the beam channel and allow for an optimized water flow of 9 l min^−1^ on each slit, enabling them to absorb more than 1200 W.

Each slit subcomponent is mounted on an *X*/*Y* stage from FMB Berlin and is equipped with absolute encoders. The openings of the slits have been selected such that they fit all IDFEs and hence reduce the overall costs.

#### 2.1.7. Vacuum window

Vacuum windows at hard X-ray beamlines serve two primary purposes. Firstly, they separate the beamline vacuum from the storage-ring vacuum. This separation is crucial for beamlines with moderate vacuum conditions or when handling pollutant or toxic samples and is also essential in incidents such as air inrushes. Secondly, vacuum windows act as high-pass filters, allowing high photon energies to pass through, while absorbing lower energies, thus reducing the heat load on downstream elements. This filtering capability makes vacuum windows unsuitable for soft X-ray beamlines.

**Figure 9:**
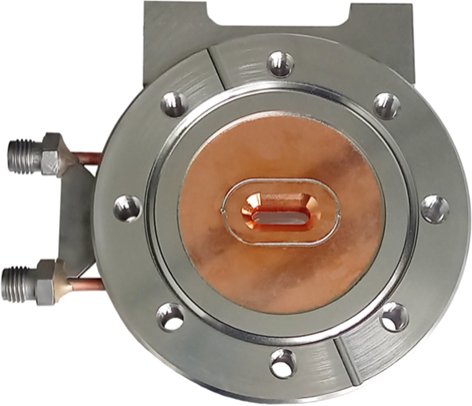
CVD diamond window for hard X-ray front ends.

For SLS 2.0, most existing chemical vapour deposition (CVD) diamond windows (Blumer *et al.*, 2006 ▸) (see Fig. 9 ▸), with thicknesses ranging from 80 to 100 µm (see Table 1 ▸), are reused. At PX-III (X06DA), the vacuum window is equipped with a 100 µm beryllium foil, as the beamline operates at energies as low as 2 keV, below the *K*-absorption edge of carbon. While the CVD diamond windows were fabricated in-house, the beryllium vacuum window was custom-manufactured by Metal Technology Co. Ltd (MTC) according to our specifications.

#### 2.1.8. Special components and configurations

Some front ends differ significantly from the standard IDFEs; these are described here for completeness.

The I-TOMCAT front end (X02SA) is entirely new, as no beamline existed in this straight during the SLS era. Also, for this front end, the CVD diamond window is placed by the pump stand after the HP slits. Additionally, we plan to install a water-cooled silicon-wedge high-power filter after the CVD diamond window and upstream of the tunnel wall.

At cSAXS (X12SA), the installation of a second set of HP slits is planned after the pump stand and the first set of HP slits. It is equipped with two tungsten blades to achieve a better-defined beam profile.

### 2.2. Bending-magnet front ends (BMFEs)

**Figure 10:**
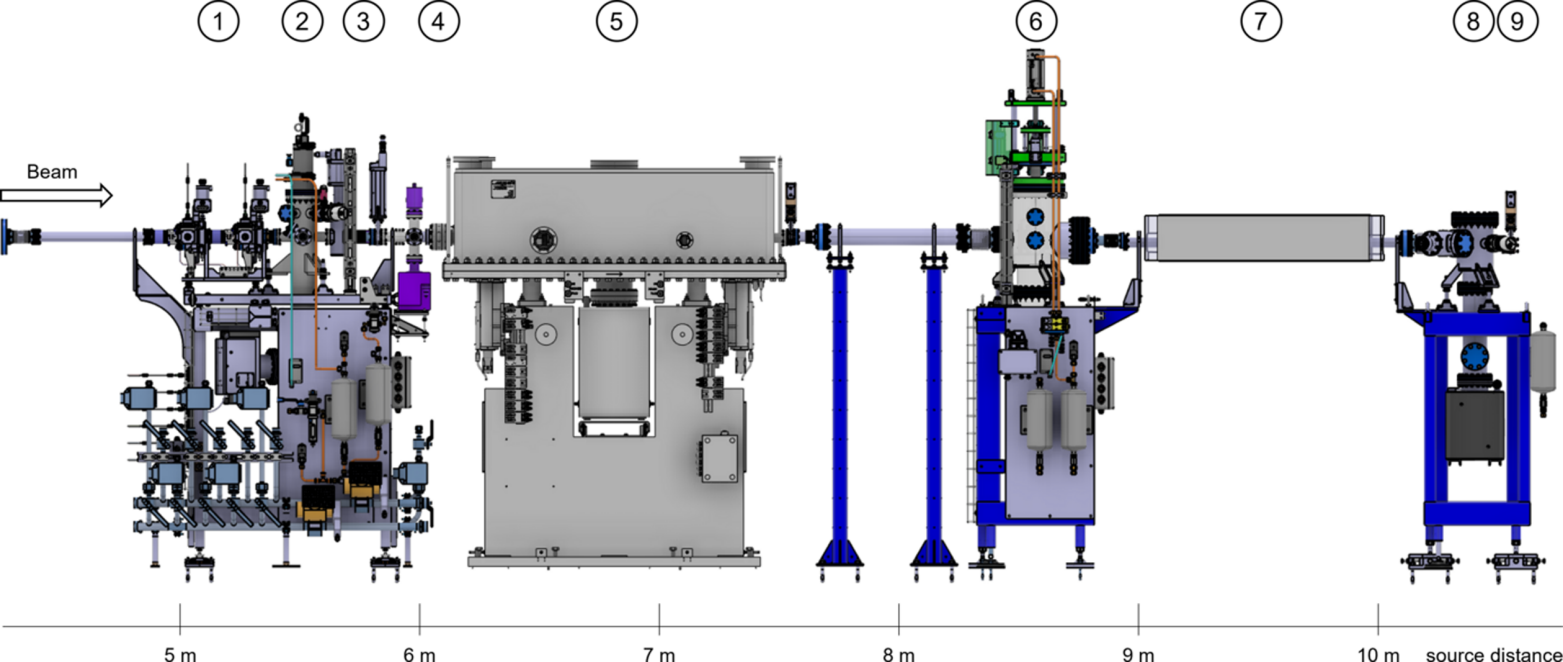
Typical bending-magnet front end (BMFE), here X06DA (PX-III). (1) Diaphragm/slits, (2) absorber, (3) gate and fast valve, (4) vacuum window with bypass, (5) collimating mirror, (6) photon shutter, (7) tunnel wall, (8) existing (SLS) after wall pump stand, (9) front-end terminating gate valve.

Fig. 10 ▸ illustrates a typical BMFE equipped with a collimating mirror. Unlike the more uniform IDFEs, BMFEs are more diversely constructed. The large bending-magnet radiation fan allows for a wider selection of beamline acceptances, and the relatively low power density permits a greater variety of equipment to be placed closer to the source, inside the front ends. Below is a list of some special cases which differ from a typical BMFE.

To obtain the lowest spectral bandwidth from their monochromators, Debye (X01DA), Optics (X05DA), PX-III (X06DA) and SuperXAS (X10DA) are equipped with a collimating mirror inside the front end. Other front ends do not include this element.

The S-TOMCAT (X02DA), Optics (X05DB) and SuperXAS (X10DA) have an additional filter installed. At S-TOMCAT, the filter is a new development for SLS 2.0 and is placed between the vacuum window and the photon shutter. At SuperXAS, the filter, reused from SLS, is installed before the mirror chamber.

At Optics (X05DA), the filter and a second set of horizontal slits are part of an optical assembly that includes a monochromator and a deflecting mirror. This assembly, previously used at SLS, is now updated to accommodate additional diagnostics.

At VUV (X04DB), due to the large acceptance angle of 8 mrad × 4 mrad (H × V), determined by the crotch absorber of the storage ring, the typical diaphragm/slits combination used for BMFEs is not implemented. Instead, the existing slits, which accommodate this acceptance, have been refurbished and reused.

#### 2.2.1. Diaphragms/slits

**Figure 11:**
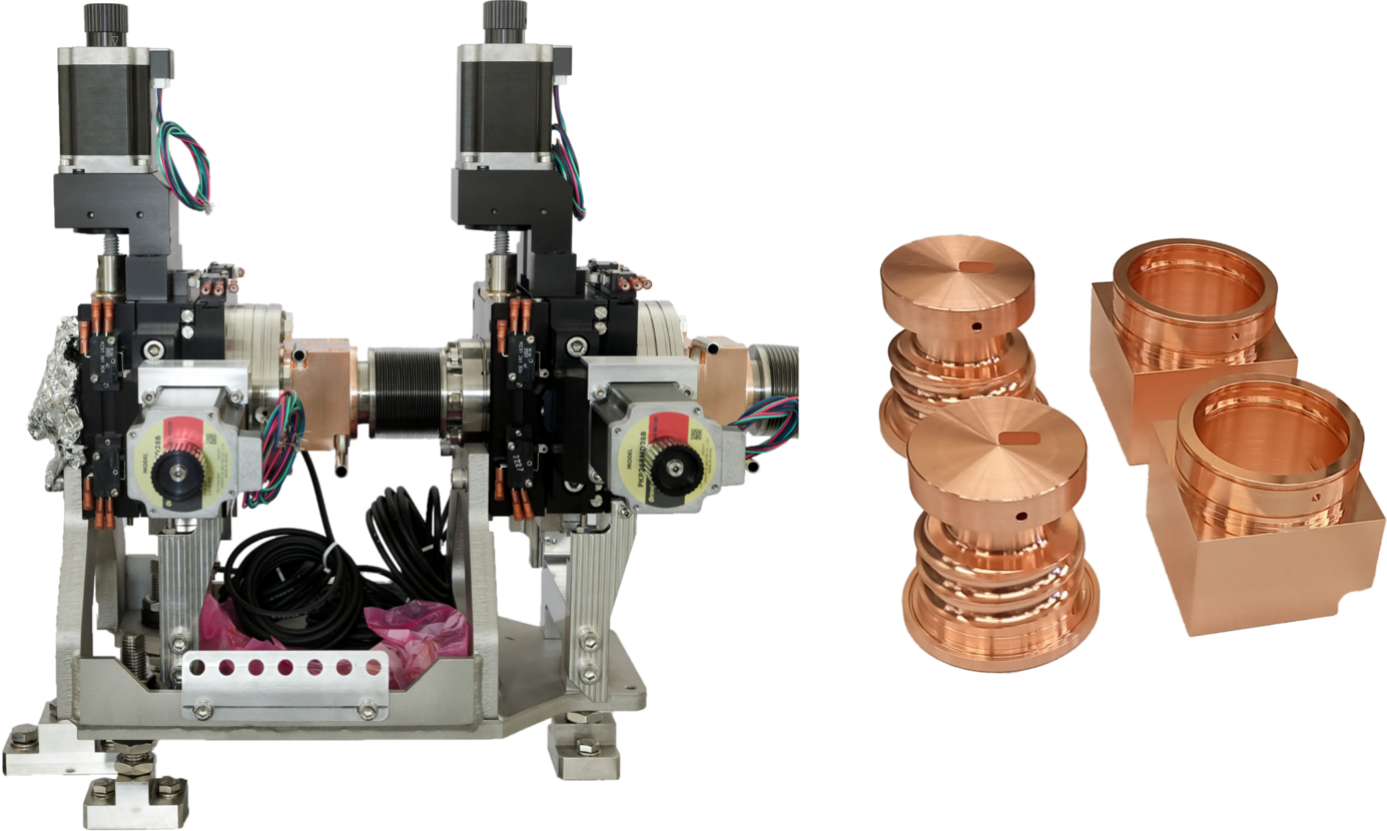
Diaphragm/slits for BMFEs, combining two functions in one device: defining the front-end acceptance (diaphragm function), and acting as slits (L-type). Left: photograph of the assembled diaphragm/slits including motion stage, absolute encoders and adjustable support. Right: photograph of the beam-defining parts prior to brazing.

Fig. 11 ▸ shows the diaphragm/slits used in BMFEs. These devices integrate the functions of both a diaphragm and slits into one unit. This space-saving design requires tailored openings for each beamline.

The diaphragm/slits are motorized with off-the-shelf *X*/*Y* stages from VAb Vacuum GmbH and are equipped with absolute encoders to realize a similar slit functionality to the HP slits used in IDFEs. The cooling system features a water channel which spirals coaxially along the beam-defining channel with 3.4 l min^−1^ for optimal cooling and minimal thermal expansion.

#### 2.2.2. Absorber

**Figure 12:**
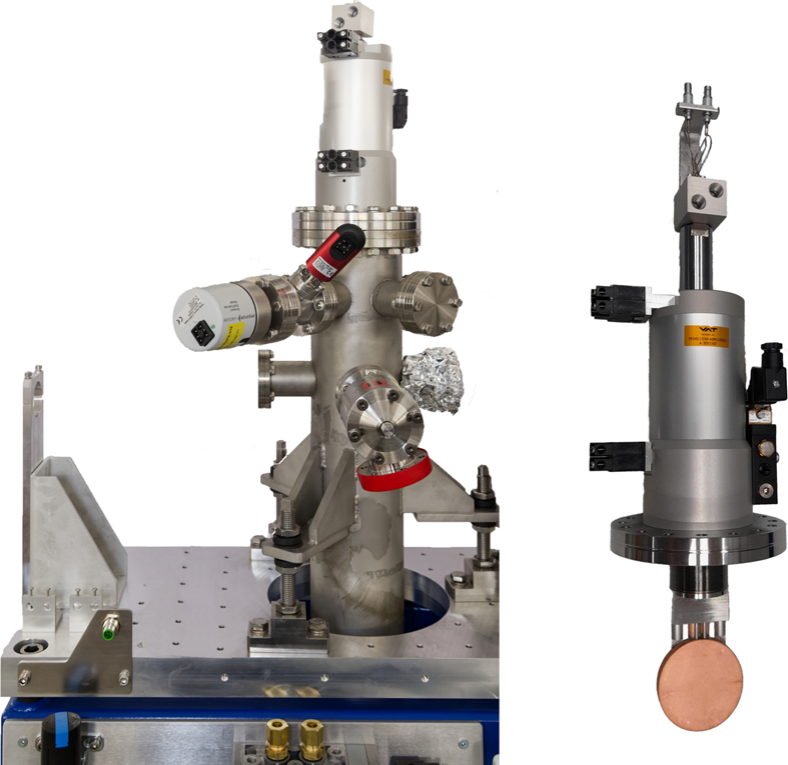
Absorber for BMFEs. Left: absorber mounted into the vacuum chamber and on support. Right: absorber insert.

Fig. 12 ▸ illustrates the absorber used in BMFEs. This component protects the gate and fast valves in case of a vacuum incident while the storage ring is operational. For SLS 2.0, the existing absorbers were replaced with commercial products from VAT, equipped with additional end switches and thermocouples to monitor the absorber’s temperature. If the water-cooled (3.3 l min^−1^) absorber overheats, the storage-ring beam is dumped to protect downstream front-end devices.

#### 2.2.3. Primary stopper

**Figure 13:**
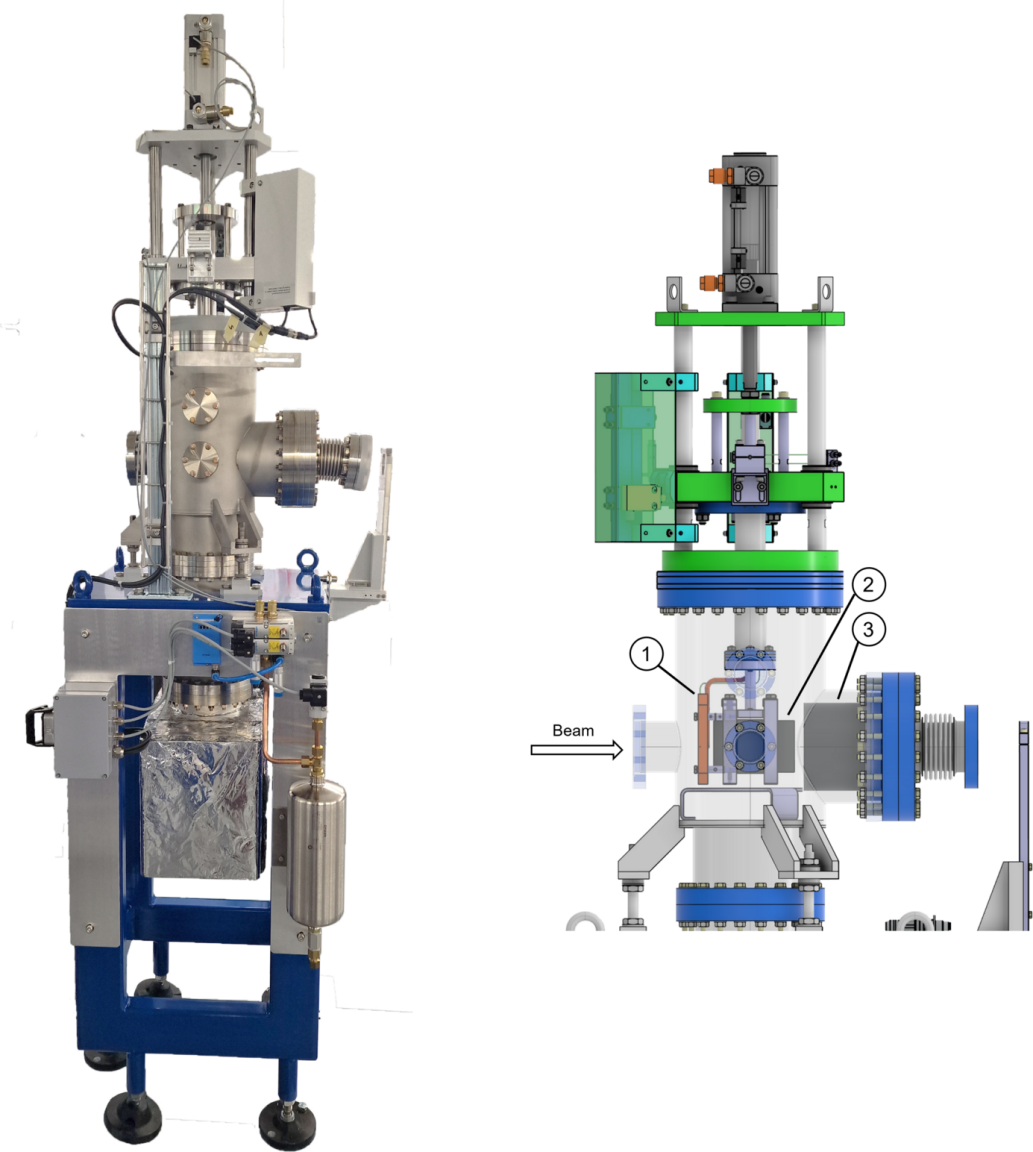
Primary stopper for BMFEs. Left: photograph of an assembled primary stopper. Right: detailed 3D model of the design. With (1) copper photon stopper, (2) tungsten beam blocker and (3) tungsten collimator.

For BMFEs, the primary stopper consists of a single device, unlike the primary stopper for insertion devices. The latter separates the functions of absorbing the synchrotron beam’s heat load and the high-energy radiation, while the former combines these functions (see Fig. 13 ▸). The synchrotron radiation is absorbed by a 12 mm-thick water-cooled (1.5 l min^−1^) copper plate (Cu-OFE), equipped with two thermocouples to trigger an escalation of the safety system in case of overheating. A tungsten alloy (Densimet G18 or equivalent) beam blocker absorbs high-energy radiation, such as *Bremsstrahlung*. Along with a collimator, also made from tungsten alloy, these components allow beamline personnel to work in downstream areas even while the storage ring is in operation.

The bending-magnet primary stoppers, reused from the existing SLS front ends, were significantly modified for SLS 2.0. All motion bellows were replaced with ones designed to last for 1 million cycles. All pneumatic components were replaced with state-of-the-art models to ensure a long lifetime. To improve the safety and reliability level, several improvements have been implemented. The existing micro-switches were replaced with certified safety end switches of two different manufacturers (diversity), mounted on two different supports (redundancy). The pneumatic control block was upgraded to a model certified for safety applications. Additionally, a fall-through protection was integrated into the design to ensure that the photon absorber and the beam blocker remain in the beam path even if the driving rod fails.

## 3. Conclusions

Currently 12 of 18 front ends for user beamlines are being refurbished and prepared for installation in late 2024 as part of a sustainable and cost-effective upgrade campaign. Each front end is tailored to the specific needs of its beamline and the new beam properties of the SLS 2.0 storage ring. All reused components are carefully updated to ensure long-term reliability and compliance with new safety standards. Novel designs were devised to enhance cooling capacity to accommodate the increased power load of the storage ring, provide greater stability required by new scientific demands, and fit the compact footprint necessitated by the existing exit ports and tunnel walls of SLS and the new lattice of the SLS 2.0 storage ring.

## Figures and Tables

**Figure 14:**
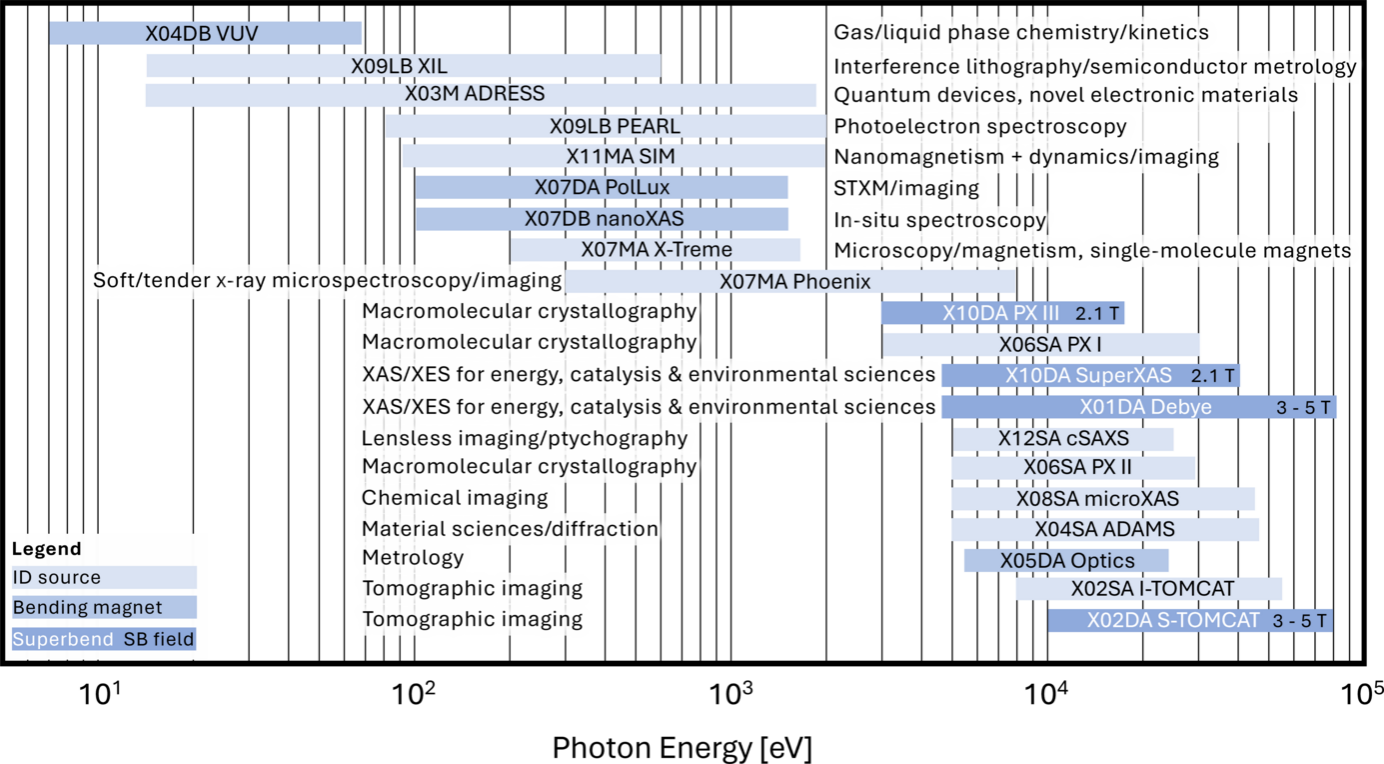
SLS 2.0 user beamlines with their energy range and scientific focus.

**Figure 15:**
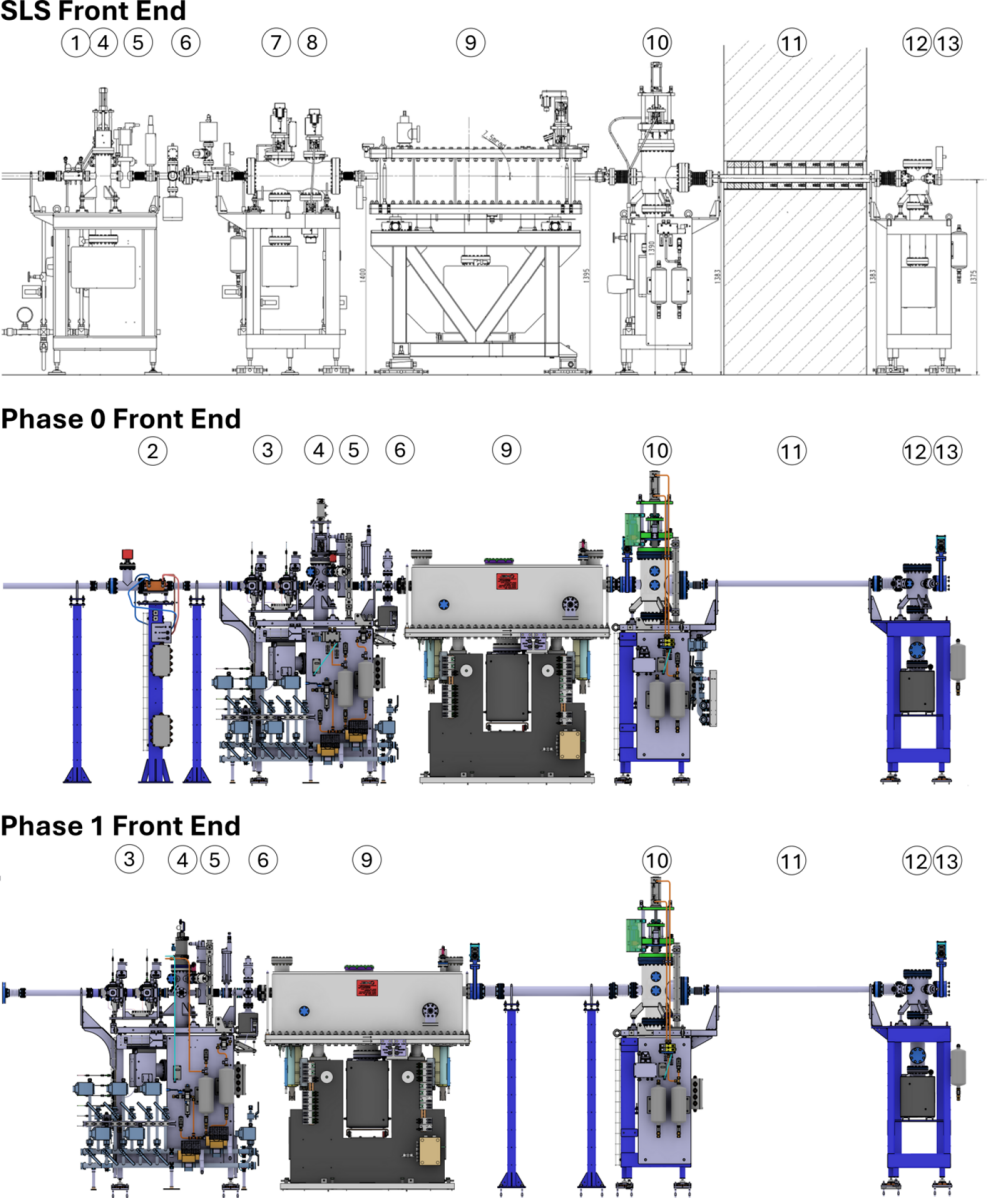
The PX-III (X06DA) front end at SLS and two different SLS 2.0 phases. (1) Diaphragm, (2) Phase 0 beam mask, (3) bending-magnet diaphragm slits combination, (4) absorber, (5) gate- and fast valve, (6) vacuum window, (7) CVD XBPM, (8) vertical slits, (9) mirror, (10) photon shutter, (11) tunnel wall, (12) pump stand, (13) front-end terminating gate valve.

**Table 1:** User front ends portfolio of SLS 2.0, with beamline acceptance and resulting power loads Power values used for the front-end design. Final values, especially for Phase 2 insertion-device beamlines, might change, since their design is not yet fully defined. In the ‘Source’ column, BM stands for bending magnet, SB superbend, U linear undulator, UE elliptical undulator, kn knot-type undulator.

Beamline	Source	Beamline acceptance (H×V) (mrad)	Power on front end (W)	Maximum power density (W mrad^−2^)	Maximum power after front end (W)	Vacuum window
X04DB VUV†‡	BM 1.4 T	8.0 × 4.0	360	160	360	None
X05DA Optics†‡	BM 1.4 T	2.0 × 0.67	100	160	50	None
X07DA PolLux‡	BM 1.4 T	1.8 × 0.79	100	160	80	None
X07DB nanoXAS‡						
X01DA Debye	SB 5 T (maximum)	1.8 × 0.79	370	580	270	0.1 mm C
X06DA PX-III						0.1 mm Be
X10DA SuperXAS						0.1 mm C
X02DA S-TOMCAT,	SB 5 T (maximum)	2.0 × 0.66	370	580	300	0.1 mm C
X02SA I-TOMCAT	U10	0.1 × 0.1	7800	48400	410	0.1 mm C
X03M ADRESS †‡	UE36kn 4 m	0.25 × 0.25	3900	24700	1240	None
X04SA ADAMS	U14	0.1 × 0.09	7600	47400	360	80 µm C
X06SA PX-I	U16	0.1 × 0.09	10000	59400	340	80 µm C
X08SA microXAS†						0.1 mm C
X10SA PX-II						80 µm C
X12SA cSAXS						0.1 mm C
X07MA Phoenix/X-Treme†‡	UE36 2 m	0.25 × 0.25	1420	12300	790	None
X09LB XIL/Pearl †‡	U70	0.36 × 0.36	610	3300	300	None
X11MA SIM‡	2× UE36kn 2 m	0.25 × 0.25	7500	15400	800	None

† Phase 2 front end (installation Q1/2026).

‡ Soft X-ray front end.

## Appendix A SLS 2.0 user beamlines

A graphical overview of the energy range and the scientific focus for each user beamline is given in Fig. 14 ▸.

## Appendix B Front-end layout comparison

A comparison of the layout of the PXIII (X06DA) front end in Phase 0 and Phase 1 as well as in its original SLS state is given in Fig. 15 ▸.
